# A Modeling Approach on Why Simple Central Pattern Generators Are Built of Irregular Neurons

**DOI:** 10.1371/journal.pone.0120314

**Published:** 2015-03-23

**Authors:** Marcelo Bussotti Reyes, Pedro Valadão Carelli, José Carlos Sartorelli, Reynaldo Daniel Pinto

**Affiliations:** 1 Instituto de Física, Universidade de São Paulo, São Paulo, Brazil; 2 Centro de Matemática, Computação e Cognição, Universidade Federal do ABC, Santo André, Brazil; 3 Departamento de Física, Universidade Federal de Pernambuco, Recife, Brazil; 4 Instituto de Física de São Carlos, Universidade de São Paulo, São Carlos, Brazil; Federal University of Rio Grande do Norte, BRAZIL

## Abstract

The crustacean pyloric Central Pattern Generator (CPG) is a nervous circuit that endogenously provides periodic motor patterns. Even after about 40 years of intensive studies, the rhythm genesis is still not rigorously understood in this CPG, mainly because it is made of neurons with irregular intrinsic activity. Using mathematical models we addressed the question of using a network of irregularly behaving elements to generate periodic oscillations, and we show some advantages of using non-periodic neurons with intrinsic behavior in the transition from bursting to tonic spiking (as found in biological pyloric CPGs) as building components. We studied two- and three-neuron model CPGs built either with Hindmarsh-Rose or with conductance-based Hodgkin-Huxley-like model neurons. By changing a model’s parameter we could span the neuron’s intrinsic dynamical behavior from slow periodic bursting to fast tonic spiking, passing through a transition where irregular bursting was observed. Two-neuron CPG, half center oscillator (HCO), was obtained for each intrinsic behavior of the neurons by coupling them with mutual symmetric synaptic inhibition. Most of these HCOs presented regular antiphasic bursting activity and the changes of the bursting frequencies was studied as a function of the inhibitory synaptic strength. Among all HCOs, those made of intrinsic irregular neurons presented a wider burst frequency range while keeping a reliable regular oscillatory (bursting) behavior. HCOs of periodic neurons tended to be either hard to change their behavior with synaptic strength variations (slow periodic burster neurons) or unable to perform a physiologically meaningful rhythm (fast tonic spiking neurons). Moreover, 3-neuron CPGs with connectivity and output similar to those of the pyloric CPG presented the same results.

## Introduction

Central Pattern Generators (CPGs) are small neural networks specialized in endogenously providing periodic and reliable patterns to generate rhythmic motor activity [1–3] such as chewing, walking, flying, breathing, etc. They work in an almost autonomous fashion in which the supervision of higher centers mostly consists in shaping or fine-tuning the patterns to a specific purpose. Studying simpler CPGs can bring some light on how larger circuits (e.g. in the brain) generate spatio-temporal patterns [4, 5]. Some CPGs produce the very similar activity in the living animal and “in vitro”, thus being especially suitable for studies in neurophysiology. One of these remarkable circuits is the pyloric CPG of the crustacean stomatogastric ganglion (STG) [6–8]. This network has been widely studied for more than 35 years and most of its physiological details are known. Several pattern generation mechanisms have been proposed [9–13] but the genesis of the motor patterns is still not rigorously understood [14].

Crustacean pyloric CPGs are extremely robust, in the sense that they have the property of keeping generating a functional pattern when subject to physiological or environmental changes [15–18]. The pyloric CPG is robust under strong changes in neuromodulation [19, 20], excitability [21], synaptic strengths [22], and temperature [23].

Nevertheless, the pyloric CPG, when submitted to environmental, physiological or modulators changes, also have the property of easily changing its activity, what is usually referred to as flexibility [24–28]. Experimental data from the pyloric circuit shows that these CPGs are highly flexible, being able to present up to 5-fold changes in their bursting frequency “in vitro” [21, 29] and 3-fold changes “in vivo” [30], while keeping their relative bursting phase of the neurons.

Reductionist attempts to understand the genesis of robust and flexible rhythms in apparently simple circuits, such as the pyloric CPG, have failed because of the complex nature of the mechanism [14]. It has been found in experiments that similar network performance is obtained from highly variable sets of network parameters [31, 32], the same results were reported using a database of models based in STG neurons [33]. These findings have been considered as evidences of the role of homeostasis in shaping intrinsic and network parameters to compensate for variability while achieving functional robustness [5]. In spite of all biophysical variability, most of the CPG motor neurons present a similar chaotic-like bursting behavior when isolated from the network [14, 34–36]. This findings suggest a relation between the intrinsic dynamical behavior of neurons and the set of rhythms that emerges as a collective property of the complex CPG. Moreover it raises a question: Why to use irregular neurons to generate rhythmic activity?

Appealing ideas such as the control of chaos [37, 38], in which the stabilization of any periodic orbit out of an infinite number of unstable periodic orbits embedded in a phase space could be reliably achieved either by slightly changing the value of some relevant control parameter or simply by applying a small perturbation, led some researchers to hypothesize that the main role of chaos in the isolated neurons is to provide a neural network with both flexibility and reliability [14, 39, 40].

Here we addressed the hypothesis about the flexibility and robustness of CPG’s built on irregular neurons. We used CPG models with two and three neurons and two types of neural models, one phenomenological Hindmarsh-Rose-like [41–44] and one realistic conductance-based Hodgkin-Huxley-like model [45, 46]. Here we adopted the amplitude of frequency changes as a function of synaptic strength as a measure of flexibility. The robustness was assessed by the standard deviation of the burst duration and the absence of non-oscillatory behavior. These choices are not unique, but they seemed to be good approximations to those complex concepts.

We analyzed how these objective measures were influenced by changes on the intrinsic excitability of neurons. We found that in order to obtain the more flexible and robust CPGs one have to tune the intrinsic parameter of the neurons in a narrow range of values were they present irregular bursting in the transition to tonic behavior and not in any other parameter range.

## Materials and Methods

We used two model neurons: a four-dimensional Hindmarsh-Rose-like model (HR4) [41, 42, 44] and a two-compartment model (HH2C) based on a twelve-dimensional Hodgkin-Huxley-like model (HH) [45–47]. Results from an improved HH2C model, inspired in previous works on the role of the hyperpolarization-activated current (I_H_) [12, 48–50], in which the I_H_ dynamics was changed are also presented.

Synaptic connections were implemented by using a first order kinetic model of neurotransmitter release [51, 52]. The integration was performed using an adaptive-step 6th order Runge-Kutta algorithm. The numerical integration error at each step was kept smaller than 10^-6^ mV. The first 10 seconds of each simulation were discarded to eliminate the transient behavior and the following 60 seconds of activity were considered for analysis.

At the end of each simulation the last state of the variables were saved and used as initial conditions for the next integration. We also compared the parameter space built by sequentially incrementing the parameters with the one built by randomly choosing both the parameters and the initial conditions of the neurons to ensure no hysteresis is present.

### Hindmarsh-Rose-like model (HR)

The HR model was chosen because it is the one of the simplest neuron models that is able to qualitatively mimic both the behavior of STG isolated neurons as well as the behavior of pair of neurons connected by mutual inhibition [44]. Furthermore, experiments have shown that this model successfully replaced a damaged neuron in the pyloric circuit [43].

The HR model we used is given by:

$$\begin{array}{ccc} \dot{x} & = & Ay-Cx^{3}+Bx^{2}-Dz+I+I_{syn}\\ \dot{y} & = & E-Fx^{2}-y-Gw\\ \dot{z} & = & \mu \left(S(x+H)-z\right)\\ \dot{w} & = & \nu (R(y+L)-Kw) \end{array}$$

where A = 1, B = 3, C = 1, D = 0.99, E = 1.01, F = 5.0128, G = 0.0278, H = 1.605, K = 0.9573, L = 1.619, *μ* = 0.0021, *ν* = 0.0009, R = 3, S = 3.966.

The x variable represents the membrane potential, while I represents a DC current injected in the neuron. The y variable represents a fast component of the total current trough the membrane and z and w are the slow components responsible for the bursts. Isyn is the dynamic postsynaptic current due to the connections. The parameter I was used to adjust the intrinsic behavior of the model from periodic spiking to chaotic bursting and finally tonic spiking.

### Hodgkin-Huxley-like model (HH2C)

Our HH2C model was based on a single compartment HH model that was first developed and then updated to better represent the ion channels and their dynamics as observed in voltage-clamp experiments with STG neurons in primary culture [45–47].

We modified this HH model in order to include deeper long lasting spontaneous hyperpolarizations, which are not only a very important characteristic of STG bursting neurons, but they also allow one to design networks using graded synaptic connections, believed to play crucial roles in CPG pattern formation [7, 53–57].

Since most works on modeling STG neurons are based in whole cell voltage clamp experiments of cultured neurons and there are not enough data to decide, without speculative assumptions, neither how many compartments are needed, nor how to segregate or how to split the many different conductances among compartments, we decided to modify the model in the simplest possible way yet capable to perform deeper hyperpolarizing behavior. Our modification consisted in splitting the different ion channel conductances of the HH2C model neuron in two compartments: an axonal compartment where the spike generating fast Na^+^ and K^+^ plus a passive leakage currents are located, and a somatic/neuropil compartment that includes all slow conductances of the model as well as the synaptic input current. The two compartments were connected by a passive electrical conductance adjusted in such a way that the bursting behavior of the model is similar to the one found in STG neurons.

The membrane potential dynamics of the two compartments are given by:

$$\begin{array}{ccc} C\frac{dV}{dt} & = & -(-I_{syn}+I_{CaT}+I_{CaS}+I_{A}+I_{KCa}+I_{H}+I_{leak}+G_{VV}\left(V-V_{Ax}\right))\\ C_{Ax}\frac{dV_{Ax}}{dt} & = & -\left(I_{Na}+I_{Kd}+I_{leakAx}+G_{VV}\left(V_{Ax}-V\right)\right) \end{array}$$

where C = 0.628 nF is the capacitance of the soma whose area is 0.628×10^-3^ cm^2^, C_Ax_ = 0.2 nF is the capacitance of the axon of area Area_Ax_ = 0.2×10^-3^ cm^2^, G_VV_ = 200 nS is the conductance coupling the two compartments. These values are based on STG neurons properties.

Besides the separation of the model in two compartments, we kept all the remaining parameters as shown by Prinz et al. [45], with the exception of the E_leak_ that was slightly decreased to produce the desired deeper hyperpolarizations.

The currents are described by

$$\begin{array}{c} I_{i}=g_{i}m_{i}^{\gamma_{i}}h_{i}^{\delta_{i}}\left(V-E_{i}\right)A_{c} \end{array}$$

where g_i_ is the maximal specific conductance: g_Na_ = 120 mS/cm^2^; g_CaT_ = 1.0 mS/cm^2^; g_CaS_ = 11.0 mS/cm^2^; g_KCa_ = 1.22 mS/cm^2^; g_Kd_ = 36 mS/cm^2^. A_c_ is the area of the corresponding compartment (soma or axon). The reversal potentials are: +50 mV for Na, -80 mV for the three potassium currents, -20 mV for I_H_, and -60 mV for I_leak_. The calcium reversal potential was calculated by the instantaneous intracellular calcium concentration and an extracellular calcium concentration [Ca]_ext_ of 3 mM, by means of the Nernst equation, at 17°C. The values for the integer exponents *γ*_*i*_ and *δ*_*i*_ are given in Table 1. The activation and inactivation variables m_i_ and h_i_ change according to:

$$\begin{array}{ccc} \tau_{m}\frac{dm}{dt} & = & m_{\infty }-m\\ \tau_{h}\frac{dh}{dt} & = & h_{\infty }-h \end{array}$$

where *τ*_*m*_, *m*_∞_, *τ*_*h*_ and *h*_∞_ are voltage-dependent, as shown in Table 1. The internal calcium concentration dynamics is given by

$$\begin{array}{c} \tau_{Ca}\frac{d\left[Ca^{+2}\right]}{dt}=-f\left(I_{CaT}+I_{CaS}\right)-\left[Ca^{+2}\right]+{\left[Ca^{+2}\right]}_{0} \end{array}$$

where *τ*_*Ca*_ = 200 ms is the Ca^+2^ buffering time constant, f = 14.96 M/nA, and [Ca^+2^]_0_ = 0.05 M is the internal calcium concentration for which there is no calcium flux through the membrane.

**Table 1:** Voltage dependence of the model currents.

	*γ*_*i*_	*δ*_*i*_	*m*_∞_	*h*_∞_	*τ*_*m*_	*τ*_*h*_
*I*_*Na*_	3	1	$\frac{1}{1+e^{\left(\frac{V+25.5}{-5.29}\right)}}$	$\frac{1}{1+e^{\left(\frac{V+48.9}{5.18}\right)}}$	$2.64-\frac{2.52}{1+e^{\left(\frac{V+120}{-25}\right)}}$	$\frac{1.34}{1+e^{\left(\frac{V+62.9}{-10}\right)}}\left(1.5+\frac{1}{1+e^{\left(\frac{V+34.9}{3.6}\right)}}\right)$
*I*_*CaT*_	3	1	$\frac{1}{1+e^{\left(\frac{V+27.1}{-7.2}\right)}}$	$\frac{1}{1+e^{\left(\frac{V+32.1}{5.5}\right)}}$	$43.4-\frac{42.6}{1+e^{\left(\frac{V+68.1}{-20.5}\right)}}$	$210-\frac{179.6}{1+e^{\left(\frac{V+55}{-16.9}\right)}}$
*I*_*CaS*_	3	1	$\frac{1}{1+e^{\left(\frac{V+33}{-8.1}\right)}}$	$\frac{1}{1+e^{\left(\frac{V+60}{6.2}\right)}}$	$2.8+\frac{14}{e^{\left(\frac{V+27}{10}\right)}+e^{\left(\frac{V+70}{-13}\right)}}$	$120+\frac{300}{e^{\left(\frac{V+55}{9}\right)}+e^{\left(\frac{V+65}{-16}\right)}}$
*I*_*A*_	3	1	$\frac{1}{1+e^{\left(\frac{V+27.2}{-8.7}\right)}}$	$\frac{1}{1+e^{\left(\frac{V+56.9}{4.9}\right)}}$	$23.2-\frac{20.8}{1+e^{\left(\frac{V+32.9}{-15.2}\right)}}$	$77.2-\frac{58.4}{1+e^{\left(\frac{V+38.9}{-26.5}\right)}}$
*I*_*K*(*Ca*)_	4	0	$\frac{\left[Ca\right]}{[Ca]+3}\left(\frac{1}{1+e^{\left(\frac{V+28.3}{-12.6}\right)}}\right)$		$180.6-\frac{150.2}{1+e^{\left(\frac{V+46}{-22.7}\right)}}$	
*I*_*Kd*_	4	0	$\frac{1}{1+e^{\left(\frac{V+12.3}{-11.8}\right)}}$		$14.4-\frac{12.8}{1+e^{\left(\frac{V+28.3}{-19.2}\right)}}$	
*I*_*H*_	1	0	$\frac{1}{1+e^{\left(\frac{V+70}{5.5}\right)}}$		$\frac{2}{e^{\left(\frac{V+169.7}{-11.6}\right)}+e^{\left(\frac{V-26.7}{14.3}\right)}}$	

The dynamical variables related to the soma currents are calculated considering the potential V, and the axon currents considering V_ax_.

**Fig 1:**
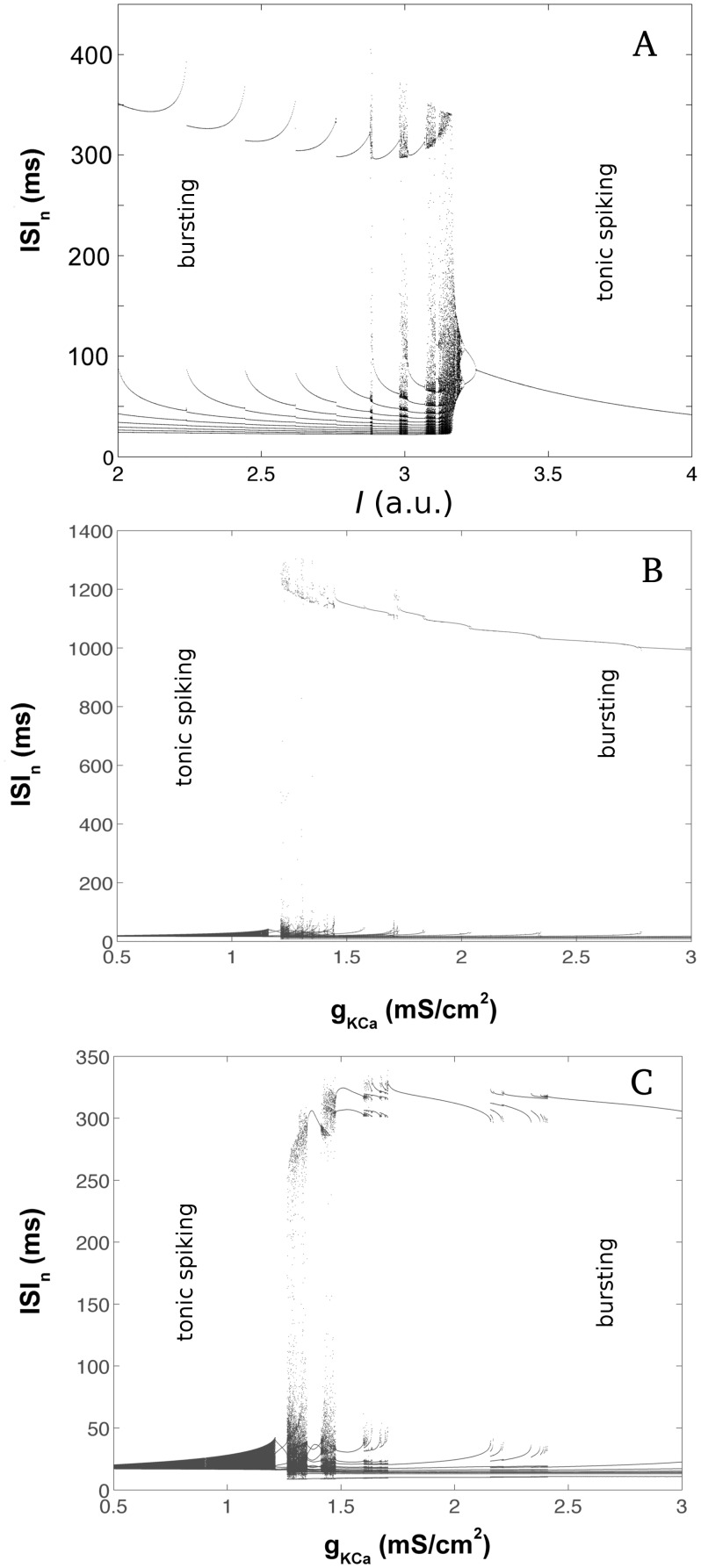
Bifurcation diagrams of the interspike intervals (ISIs) as a function of the intrinsic parameter in the regions that contained the transition from bursting to tonic spiking behavior. (A) HR model neuron: up to I∼3.1 the number of spikes per burst increased with I in a period adding bifurcation; for I>3.15 the neuron was tonic. (B) The HH2C model presented tonic behavior for values of the intrinsic parameter g_KCa_<1.21, while for g_KCa_>1.21 a bursting behavior was observed. For g_KCa_>1.45 an inverse period adding bifurcation took place. (C) HHIH model: as in the HH2C model the transition from bursting to tonic behavior happened when the control parameter was decreased.

In this model the calcium-dependent potassium conductance g_KCa_ was used to control the level of excitation of the neurons because it regulates the outflow of potassium during the spiking onset, thus it was the intrinsic parameter for the HH-based model CPGs. Varying this conductance the neuron electrical activity could be smoothly changed from rhythmically bursting to tonic spiking (Fig. 1).

### Hodgkin-Huxley-like model with increased hyperpolarization-activated current (HHIH)

The HHIH model was obtained from a modification of our previous HH2C model that consisted in changing the dynamics and increasing the value of the hyperpolarization activated current I_H_. Several facts guided this modification. First, several studies show that this conductance is important in rhythm generation [12, 48, 50]. Also, differenty from all other original HH model conductances [45] that were based on voltage-clamp data from isolated stomatogastric neurons [46, 47], the dynamics of I_H_ conductance, was taken from guinea pig thalamic relay neurons [58]. Finally, we were also inspired in previous experimental results [49] and consisted in the intensification of the I_H_ conductance and also in making it active for more depolarized potentials.

The modification were: the conductance and the reversal potential were increased (g_H_ = 0.5 mS/cm^2^; V_rev_ = 20mV) and the activation current function was slightly displaced 5mV towards more depolarized values. The modifications were made to intensify the current and to make it active for more depolarized potentials, inspired in previous experimental results [49].

The parameters for the HHIH model are: E_H_ = 20 mV, g_H_ = 0.5 and

$$\begin{array}{c} m_{\infty }^{H}=\frac{1}{1+\text{exp}\left(\frac{V+70}{5.5}\right)} \end{array}$$

All the remaining equations are the same as in the HH2C model. In the HHIH model the calcium-dependent potassium conductance g_KCa_ was also chosen as the intrinsic parameter for the model CPGs.

### Chemical synapses

The model neurons were connected by chemical synapses implemented using a model that mimics the release and the diffusion of the neurotransmitter in the synaptic cleft according to a simple first order kinetic description [51, 52]. The postsynaptic current is given by:

$$\begin{array}{c} I_{syn}=g_{syn}S\left(t\right)\left(V_{rev}-V_{pos}\left(t\right)\right) \end{array}$$

where g_syn_ is the maximal synaptic conductance, V_rev_ is the reversal potential for the synapse, and V_pos_ is the membrane potential of the post-synaptic cell. The dynamical variable S(t) is the voltage-dependent activation that mimics the kinetics release and the binding of the neurotransmitter at the synaptic cleft, and it is given by

$$\begin{array}{c} \left(1-S_{\infty }\left(V_{pre}\right)\right)\;\tau_{S}\frac{dS(t)}{dt}=\left(S_{\infty }\left(V_{pre}\right)-S\left(t\right)\right) \end{array}$$

where S_∞_ is given by

$$\begin{array}{c} S_{\infty }\left(V_{pre}\right)=\left\{\begin{array}{cc} \tanh\left(\frac{V_{pre}\left(t\right)-V_{thres}}{V_{slope}}\right) & ,\text{if}\;V_{pre}>V_{thres}\\ 0 & ,\;\text{otherwise} \end{array}\right. \end{array}$$

Table 2 shows the synaptic parameters used in the simulations for all models.

**Table 2:** Synaptic parameters used for the simulations with the HR, HH2C and HHIH models.

Parameter	HR (a.u.)	HH (2C and IH) (mV)
*V*_*thres*_	-0.8	-45
*V*_*slope*_	2.0	40
*V*_*rev*_	-1.58	-80

## Results

### Intrinsic properties of the model neurons

We characterize the intrinsic behavior of the model neurons by plotting series of interspike intervals (ISIs) as a function of the intrinsic parameter. The bifurcation diagrams show many qualitatively different behaviors the models present as the intrinsic parameter is changed (Fig. 1). In spite of each model’s particularities, all of them showed a similar sequence of dynamical states: they present a region where a period-adding bifurcation is observed and a transition from periodic bursting to tonic spiking. However, in the HR and HHIH models the transition corresponds to a band of chaotic behavior with irregular number of spikes/burst and burst duration, while in the HH2C model the transition from periodic bursting to tonic spiking is a point of accumulation of the period-adding bifurcation in which the number of spikes/burst successively increases toward infinity and the tonic behavior takes place.

### Half-center oscillators

**Fig 2:**
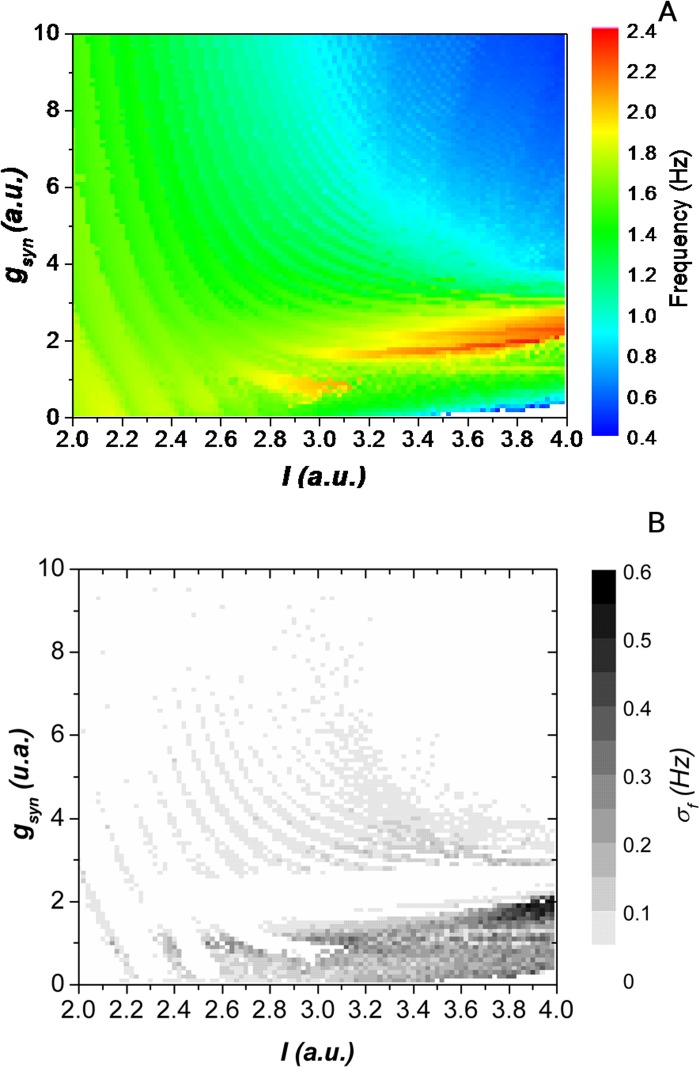
Characterization of the dynamic as a funtion of the intrinsic current and the synaptic strengh. (A) Parameter space of the bursting frequency for a two-HR model neurons half-center oscillator. The parameter I (horizontal axis) represents the intrinsic behavior of the neurons and the parameter g_syn_ (vertical axis) represents the maximal synaptic conductance between neurons. (B) Standard deviation of the bursting frequency for the same parameter space. The network is unstable for small values of the synaptic coupling when neurons become intrinsically more excited (tonic).

The parameter space for the HR model is shown in Fig. 2, where we observe that when the neurons are intrinsic periodic bursters (lower values of I) the frequency of the network is very close to the frequency of the isolated neuron and it is almost insensitive to changes in the synaptic strength.

On the other hand, when the neurons are intrinsically tonic (higher values of I) the rhythm of the network is substantially changed by the synaptic connection. One can observe a 5-fold modification in the rhythm, which goes from 0.5 Hz (when neurons are strongly connected) to 2,5 Hz (for lower synaptic coupling) in agreement with what is experimentally observed [21, 29, 30].

**Fig 3:**
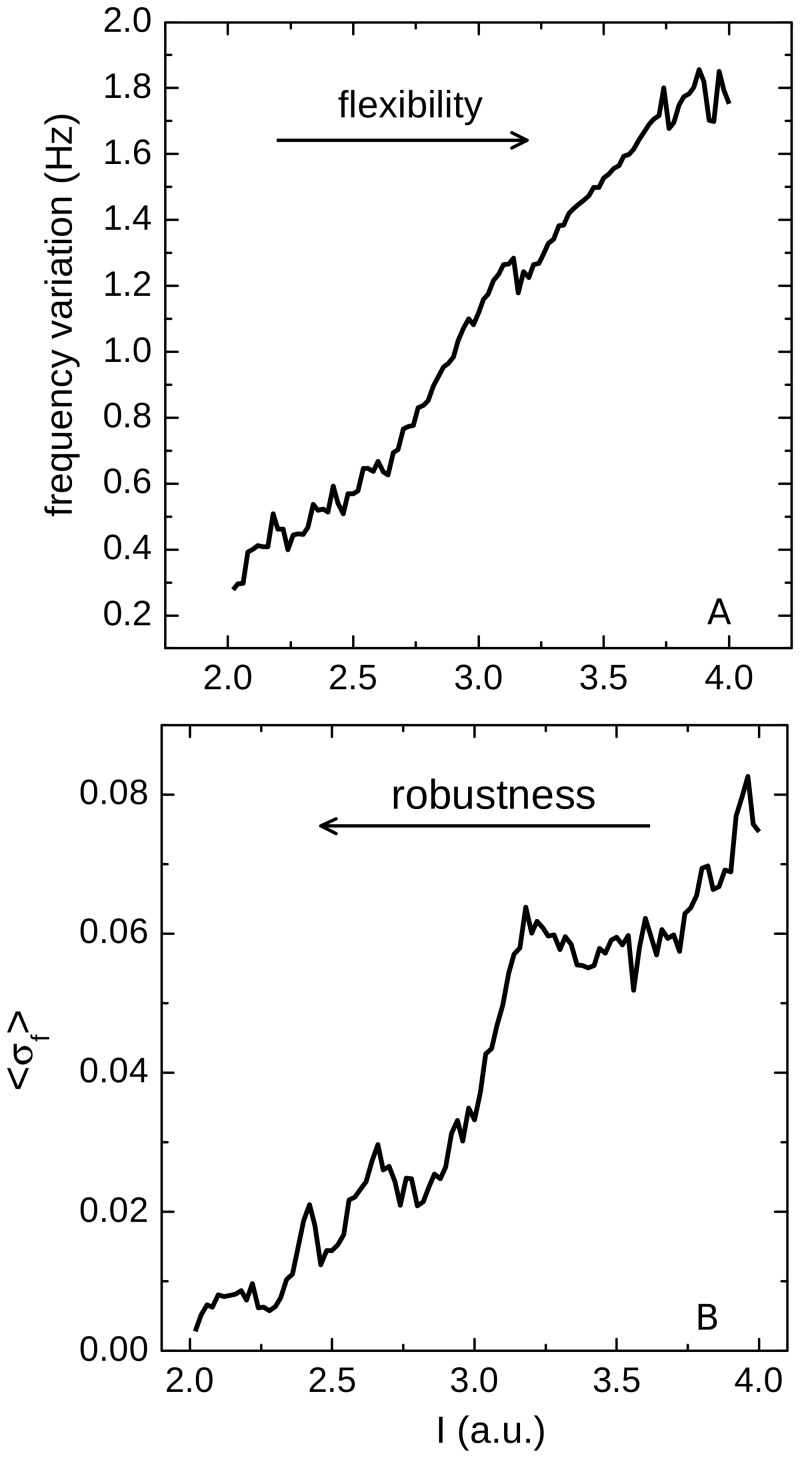
Flexibility and robustness of the network. (A) The difference between the maximal and the minimal frequencies the GPG reaches for the whole set of synaptic conductances as a function of the intrinsic parameter I. (B) The mean standard deviation of the bursting frequency (averaged over all the values of the synaptic coupling) as a function of the intrinsic parameter I. From (A) we can infer that the flexibility of the CPG increases with I, since the range of frequencies that the same neurons are able to generate is also increasing. However, in (B) we observe that the behavior of the CPGs becomes more irregular as we increase I, as it can be seen by the increase in the average standard deviation of the bursting frequency.

We inferred the network flexibility for a given value of I by computing the difference between the maximal and the minimal bursting frequency the network reached for the whole set of synaptic coupling (Fig. 3A). The robustness of the CPG for a given I was estimated by observing the average standard deviation of the bursting frequency over the synaptic strength (Fig. 3B): if the rhythm becomes irregular and the CPG fails in functionality, the bursting frequency will present large variations that will be revealed by the average of the standard deviations; conversely a robust CPG will generate periodic rhythms over all the values of the synaptic strength and will present only small standard deviations of its bursting frequencies.

In this context the more tonic were the neurons intrinsically, the more flexible was the network, as shown in Fig. 3A. However, the robustness of the CPG decreases as the neurons go from bursting to tonic, i.e., as we increased I (Fig. 3B). As the neurons became intrinsically more tonic, there was a stronger competition for firing, and the rhythm became unstable in a larger area over the parameter space as it can be seen in Fig. 2B. Moreover, when neurons were intrinsically tonic, for small synaptic strengths the network behavior is non oscillatory (white regions in Fig. 2A).

Based on these observations we argue that the best balance between flexibility and robustness is achieved when the neurons are in the transition from bursting to tonic behavior. This is the range where the model neurons present chaotic behavior when isolated.

In order to verify if these results were model-independent, we repeated the same analysis with CPGs built with HH2C model neurons, which were originated from electrophysiological measurements of STG cultured neurons.

**Fig 4:**
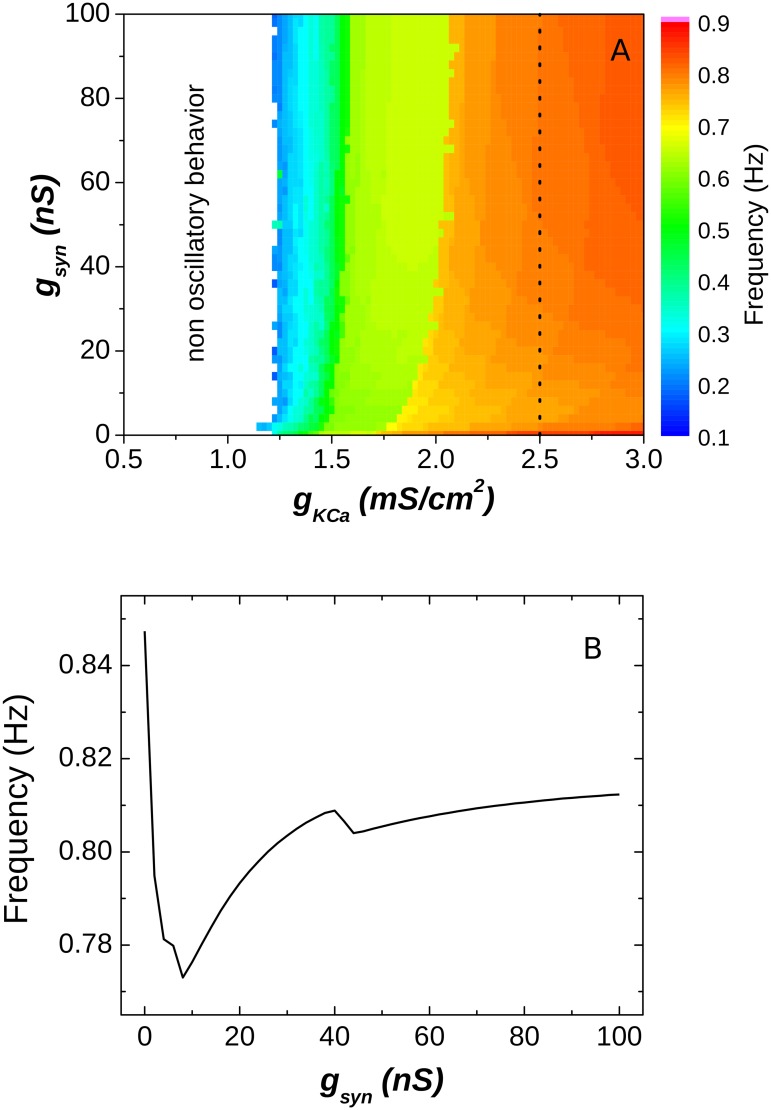
Analysis of the dynamics of the HH2C-based model. (A) Parameter space for the CPG. For smaller values of the intrinsic parameter the neurons behave tonically and do not provide usable CPGs (white region). (B) The frequency dependence on the synaptic strength for the line showed in (A) (g_KCa_ = 2.5 mS/cm^2^).

To obtain the parameter space of the HH2C-based CPGs (Fig. 4A), we used the calcium-dependent potassium conductance (g_KCa_) as the intrinsic parameter while keeping the synaptic strength (g_syn_) as the synaptic parameter.

Fig. 4A shows that these CPGs are rather insensitive to changes in the synaptic conductance, no matter what was the intrinsic behavior of the neurons. Moreover, the changes in the frequency as a function of g_syn_ (shown in Fig. 4B) occurred in the opposite direction than the observed in experiments [12]. Furthermore, the CPG stops the rhythm generation when the neurons intrinsic behavior is tonic (g_KCa_≤1.25). So, in spite of the stronger biological appeal of the HH2C in comparison to the HR model, it was not able to provide a realistic dynamical repertory of the biological CPGs.

Such discrepancy might be attributed to two factors: the model neuron was extremely sensitive to current injection, easily going to a silent state while inhibited; the model present a weak post inhibitory rebound, meaning that the activity after the release from inhibition is not increased in comparison to the ordinary activity.

One of the reasons for the HH2C model be very sensitive to inhibition is that it presents a small overall conductance during hyperpolarization, so a small current is enough to keep the neuron in the silent state. Based on these observations and in a few previous modeling and experimental works, we propose a way to improve the behavior of the model, as shown in the next section.

### Increasing the effect of I_H_ conductance in the HH model

**Fig 5:**
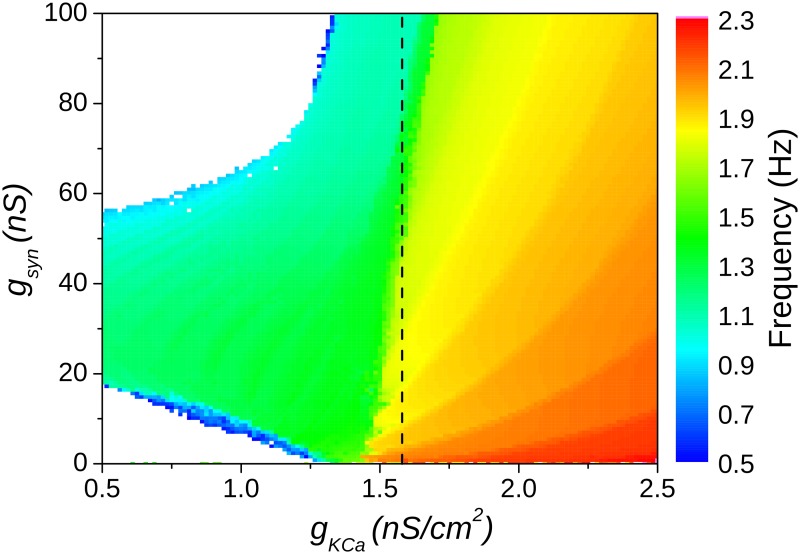
Parameter space of the burst frequency as a function of the g_KCa_ and g_syn_ for the HHIH-based CPG model. The burst frequency has its greatest gradient in the region of the transition between bursting to tonic intrinsic behavior as pointed by the dashed line.

The parameter space for the HHIH-based CPG model is shown in Fig. 5. The alteration in the I_H_ conductance corrected the dependence of the CPG frequency on the synaptic strength g_syn_, meaning that the frequency of oscillation now decreased as g_syn_ was increased, as observed for the HR model and in experiments. Furthermore, the frequency gradient increased, and so the flexibility of the CPG. Finally, the changes in I_H_ also provided the network with more stability, since the network oscillatory behavior is stable for a larger area of the parameter space.

It is worthy noting that there was a range of values of the intrinsic parameter g_KCa_ close to the transition from bursting to tonic intrinsic behavior in which the CPG had the best flexibility and it was also robust, as pointed out in Fig. 5 (the dashed line).

### A three-neurons model CPG

**Fig 6:**
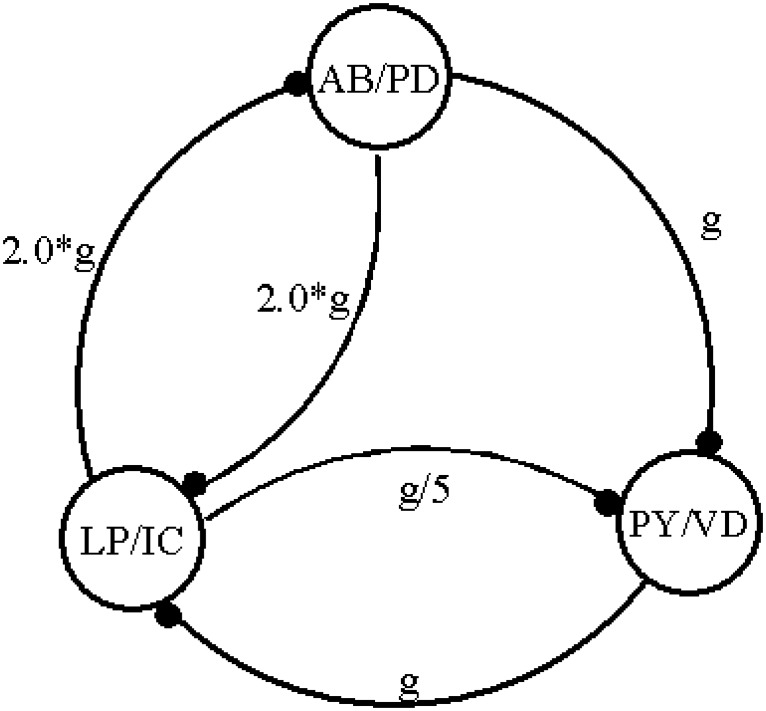
Scheme of the three phases CPG model. The connections between neurons represent the inhibitory synapses. In each synapse it is shown the value of the relative synaptic strength.

We also simulated some more realistic model CPGs to mimic the pyloric activity of the spiny lobster Panulirus interruptus. The scheme of the network is shown in Fig. 6 and it is based on the fact that food is filtered/pumped in a cycle that always preserve the same firing sequence: a cycle starts with the firing of the group formed by the Lateral Pyloric (LP) and the Inferior Cardiac (IC) neurons, on the sequence the Ventricular Dilator (VD) and the 8 Pyloric (PY) neurons fire, finally the cycle is reset by the firing of the pacemaker group formed by the Anterior Burster (AB) and the two Pyloric Dilator (PD) neurons [7, 33, 59]. Since inside each group the neurons fire with very small phase differences, in our model pyloric CPG we represented each group by a single model neuron. We also considered that all neurons have the same intrinsic properties, however we used different synaptic strengths to include the fact that in the real CPG some connections are stronger than others.

**Fig 7:**
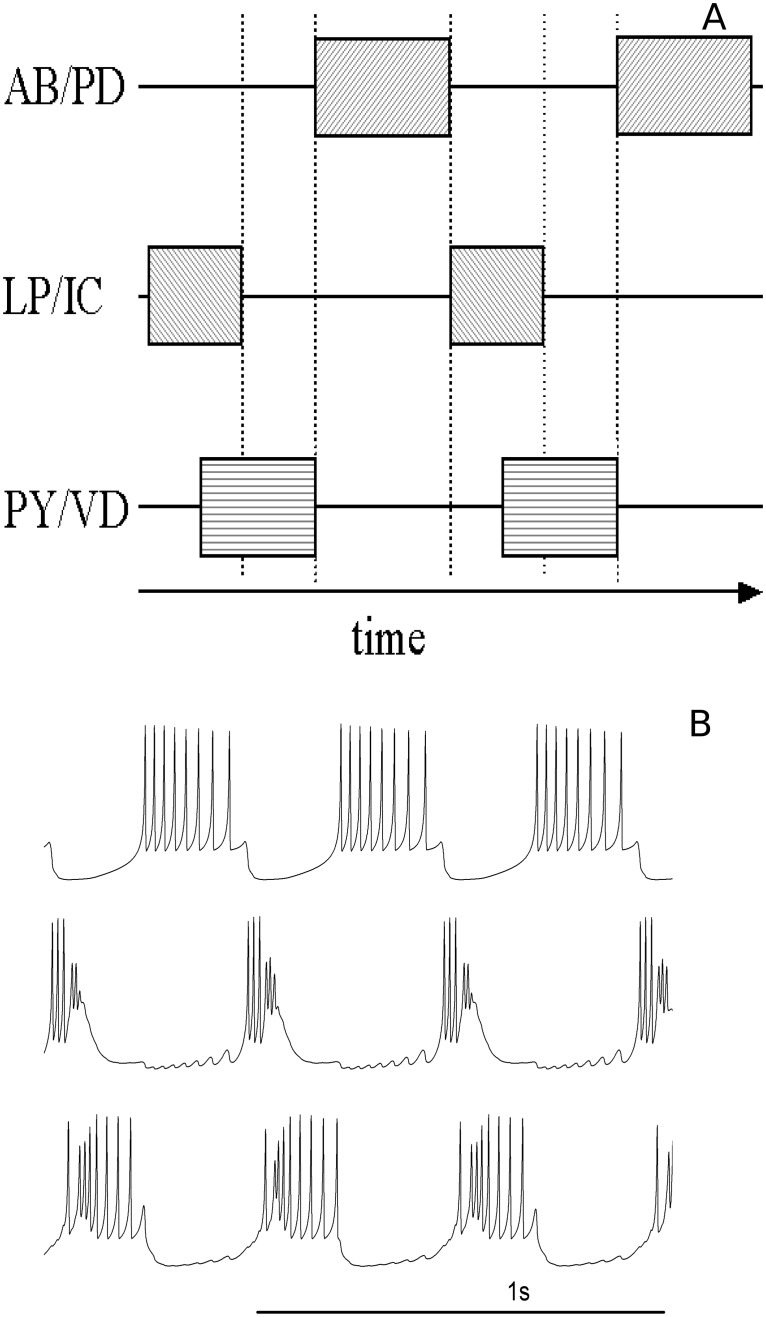
Dynamics of the 3-neuron model CPG. (A) Phase diagram showing the sequence of bursts (grey boxes) and (B) example of time series for the HR-based 3-neuron CPG. LP/IC and PY/VD bursts are superimposed as usually found in experiments. The PY/VD burst is interrupted by AB/PD and the cycle restarts.

In Fig. 7 we show the typical phase lags observed in our simulated model CPGs, as well as the respective temporal series obtained in a HR-based 3-neuron CPG.

**Fig 8:**
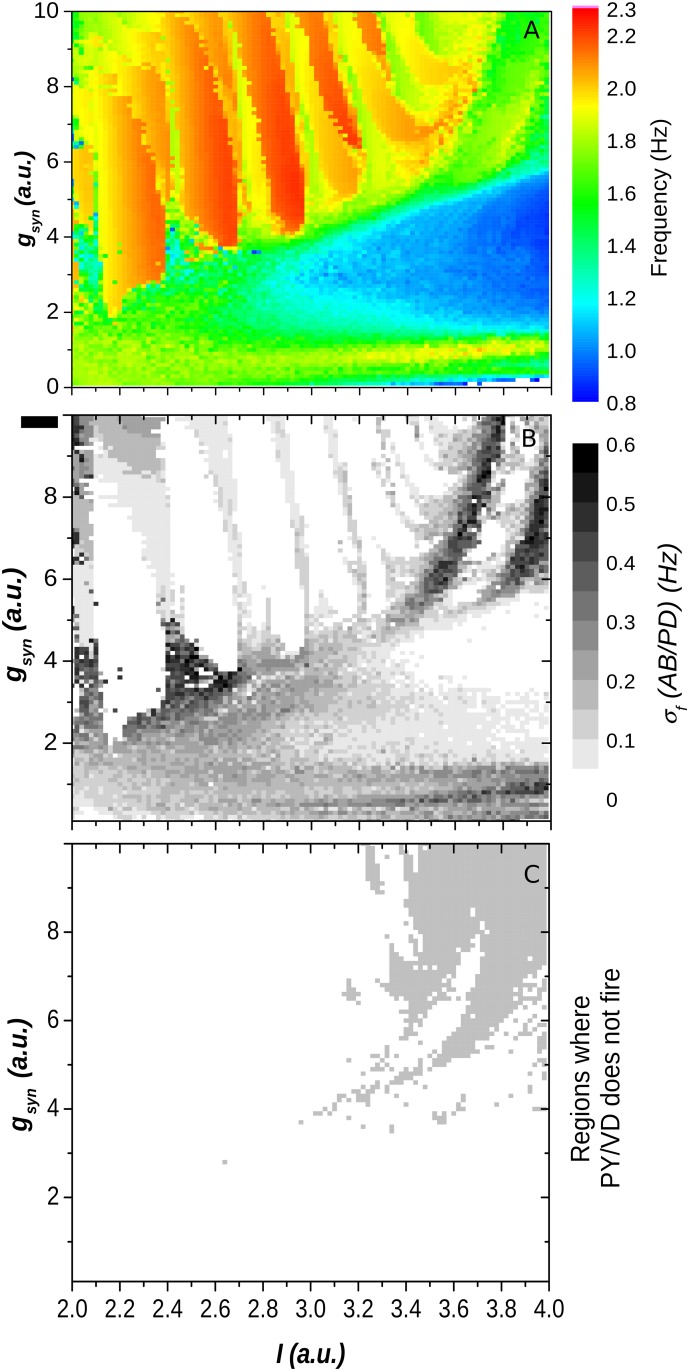
Parameter spaces as function of I and g_syn_ for a HR-based 3-neuron model CPG. (A) Bursting frequency for the AB/PD model neuron. (A) Standard deviation of the bursting frequency for the same neuron. (C) The grey regions correspond to sets of parameters where the CPG completely loses its robustness: the LP/IC neuron does not fire during the activity of the network.

The parameter spaces obtained as function of I and g_syn_ for the HR-based 3-neuron model CPG are shown in Fig. 8. For values of g_syn_<3 the results were similar to the ones obtained for the half-center oscillators: a change from 1Hz to 2Hz in the CPG frequency with the increase of g_syn_ is observed for values of the intrinsic parameter I∼3.1 (Fig. 8A) which is in the transition from bursting to tonic behavior where the model neurons are intrinsically chaotic. Around I∼3.1, and again considering only the region of the parameter space where g_syn_<3, are also found the smaller values of the standard deviation of the CPG frequencies computed from the time series of the AB/PD model neuron (Fig. 8B). For smaller values of I, where the neurons are intrinsically periodic bursters, the frequency is less sensitive to changes in g_syn_ so the CPGs are less flexible; conversely, for bigger values of the parameter I, where the neurons are intrinsically tonic, the CPG lost its robustness as it can be seen by the larger standard deviations of the CPG frequencies (as shown in Fig. 8B). For values of g_syn_>3 the results are strongly biased by the limitations of the HR model concerning the strong PIR. In these region we found several non-physiological behaviors: the presence of a series of Arnold tongues that correspond to high bursting frequencies that are very stable as it can be seen in the white regions of Fig. 8B, and a reverse dependence of the CPG frequency on g_syn_.

This discrepancies for higher values of the synaptic conductance can be understood by considering that the bursting genesis in the HR model is provided by a slow adaptation current that depends linearly on the membrane potential, instead of presenting a sigmoidal saturation as observed in biological neurons [42]. With this non-saturation characteristic of the post-inhibitory rebound (PIR) it is difficult to obtain patterns that present duty cycles much smaller than 50%, specially when a strong inhibition enhances the PIR response of the neurons. This can be seen in the region shown in Fig. 8C where the strong PIR presented by the AB/PD and VD/PY neurons prevent the LP/IC neuron of firing, disrupting the three phase rhythm of the CPG.

**Fig 9:**
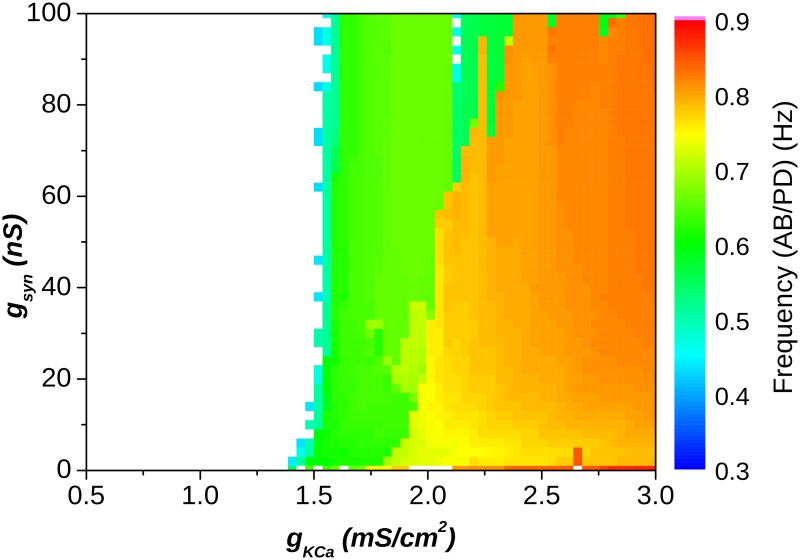
Parameter space of the bursting frequency as a function of I and g_syn_ for the HH2C-based 3-neuron CPG model. For lower values of g_KCa_ the CPG do not provide a three phase rhythm (blank region).

The parameter space obtained for a HH2C-based 3-neuron model CPG is shown in Fig. 9. In this case we observed that either no rhythm is produced by the CPG (when the neurons are set in a range of the intrinsic parameter g_KCa_ where they are intrinsically tonic), or the CPGs present almost no flexibility to changes in the synaptic conductance (for g_KCa_ in a range that corresponds to intrinsic bursting behavior).

**Fig 10:**
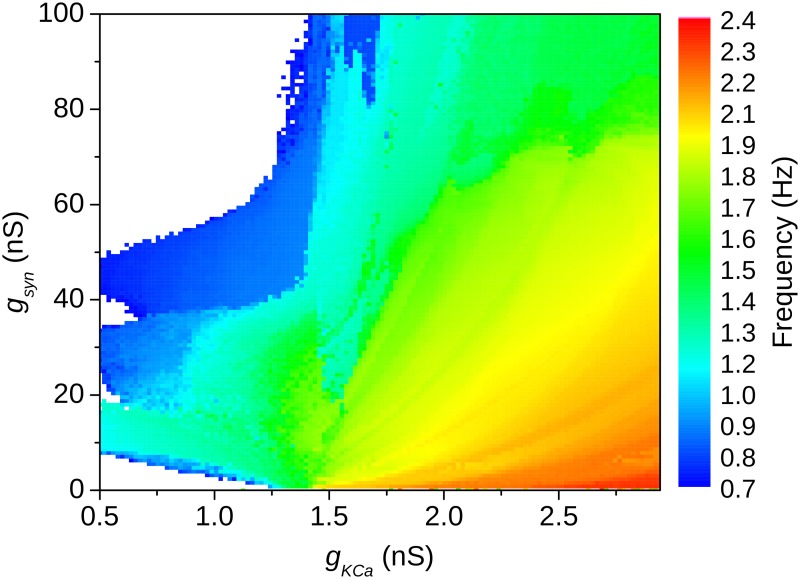
Parameter space of the bursting frequency as a function of g_KCa_ and g_syn_ for the HHIH-based 3-neuron CPG model. In comparison to the parameter space of the HH2C-based CPG the region that corresponds to bursting behavior is enlarged and a greater frequency variation is obtained in the region of g_KCa_ close to the transition between bursting to tonic behavior.

We also studied the parameter space obtained from model CPGs based on our HHIH model neurons, where we increased the influence of the I_H_ current. The results are shown in Fig. 10. Similarly to what happened with the half-center oscillator the modification brought some flexibility to our three-neurons CPG model, and the area of the parameter space where the network produces a three phase rhythm was also increased.

## Discussion

One of the main questions in neuroscience is to identify to which degree one can infer the behavior of a neural network just by investigating the isolated behavior of its neurons and the sinaptic connectivity. Not only the dynamical properties of the neurons are quite complex but the neuromodulation produces yet another layer of complexity, by changing both the intrinsic parameter of the neurons and their connectivity [60]. We tried to shed some light into this question by simulating the behavior of two- and three-neuron networks, investigating their output as the intrinsic dynamics of the neurons and the synaptic strength are changed. Our investigations reveal the mechanism and the key elements for the proper establishment of the rhythmic oscillations and its modulation by the synaptic strength.

Using reduced neural networks to investigate the dynamical properties neurons has been a successful approach to uncover features that would be hidden if the neural models were not inserted in a network. This is specially useful for understanding small GPG circuits where the number of neurons is reduced and hence the role of each individual cell is important. Connecting a neural model in a, network can be interpreted as a challenge to the model, and might reveal possible weaknesses. We believe that was the case here. A very important model (HH2C) could be improved because of a “dynamical test” under which it was subjected.

For a given value of the models intrinsic parameter we tried to assess how flexible that CPG is by changing the synaptic maximum conductance and measuring the range of bursting frequencies obtained, a common way of investigating the dynamical propperties of the CPGs [21, 29]. We evaluated the stability of a CPG through two main aspects: if a proper bursting rithms was established for all sinaptic strengths and and if the rithm had a low standard deviation of the bursting period. Even though a CPG does not requre a great amount of periodicity as for example can be obtained with computational simulations, a fair degree of periodicity is observed in biological CPGs, even when important parameters are changed [22]. Furthermore, CPGs are attached to muscles whose activation depends on a sustained activity of these neurons, and hence the muscular activity can be affected if a certain level of stability is not achieved.

### Half-Center Oscillators

For HCO based on the phenomenological HR neuron model the best relation between flexibility and robustness was obtained when the intrinsic parameter of the neurons was set in a region close to the transition between bursting and tonic spiking behaviors, as it was found in neurons isolated from the biological CPGs [36].

The original HH-like model was not able to produce half-center oscillations when the neurons were set in intrinsically tonic spiking state. Moreover, in their intrinsic bursting region they were not flexible (the frequency was not sensitive to synaptic changes). This failure in providing good models of half-center oscillators clearly shows that sometimes a successful model of an isolated neuron is not able to mimic the collective dynamics, because of the complex nature of the CPG.

We modified the HH model by changing its I_H_ conductance for experimental and theoretical reasons main reasons (see methods session). The CPGs with the resulting (HHIH) model presented three main improvements. A larger area of the parameter space became functionally meaningful, so the robustness was increased; The range of frequencies of the CPG when the synaptic parameter is changed also increased, adding flexibility to the model; It corrected the gradient of the frequency as a function of the synaptic conductance was corrected (it was reversed in the CPGs built with the original HH model).

In the improved HH model, the flexibility was maximum around the transition from bursting to tonic spiking intrinsic behavior. CPGs with tonic spiking intrinsic neurons were also less robust than those with bursting intrinsic neurons.

According to our results, when the model neurons intrinsic behavior is set to regular bursting they are able to establish anti-phase oscillations with minimal synaptic current.

If the neurons do not have a strong PIR, as soon as they achieve the anti-phase oscillatory regime, the bursting frequency is no longer influenced by changes of the synaptic conductance. On the other hand, if the neurons are intrinsically tonic, one neuron will keep firing until the other neuron escapes from the inhibition (due to I_H_ current, for example) and starts to fire. If the neurons have a weak PIR the CPG will become unstable or will not produce any oscillations.

### Three-neurons CPGs

We also presented some results obtained from 3-neurons CPG, built either with HR or HHIH models, to provide a three phase rhythm, similar to the one found in the pyloric circuit of crustaceans.

Similarly to the results obtained for HCOs, in 3-neurons model CPGs, the parameter ranges that showed the best balance between flexibility and robustness were those corresponding to intrinsic behavior in the transition from bursting to tonic spiking, the same regions where the chaotic behavior can be found in the isolated neuron models. This is again in agreement with the observation of chaotic-like dynamics in the behavior of isolated biological neurons [14, 34, 35].

Our previous discussion of the role of the PIR should be also valid for CPGs made by three or more neurons, since the competition for firing also holds. Evidently, these networks exhibit much richer behavior, since there is a stronger dependence on the connectivity. However, the general oscillating properties can still be revealed with our analysis.

### Presence of chaotic-like behavior in models and biological neurons

When pyloric STG neurons (with the exception of the AB) are isolated from the circuit, they usually behave irregularly, presenting chaotic-like bursting behavior, close to a transition to tonic spiking [14, 34–36]. The hypothesis that the main role of chaos in the isolated neurons is to provide a CPG with both flexibility and reliability is an interesting theoretical framework. Unfortunately, it only applies rigorously to isolated chaotic systems and it is challenging to show that these arguments can be extended to systems made of two or more coupled chaotic oscillators, particularly when they are strongly connected, as it is the case of the neurons of a CPG.

Here we showed that the ranges in the intrinsic parameter of the models that correspond to the best CPGs, regarding our measures of flexibility and robustness, are those where the isolated models are near a transition from bursting to tonic spiking, where a range of complex behaviors can be easily found: windows of periodic behavior, spike adding transitions, and also chaotic behavior.

However, we did not find any direct relation between the presence of chaotic behavior (at a specific values of the intrinsic parameters) and special increases on the flexibility (robustness) of the CPGs. According to our results, as the neuron models were tuned from periodic bursting to tonic spiking, the flexibility increased smoothly.

Instead of being an indicator of the role of chaos in CPGs, the fact that we always find irregular, chaotic-like behavior, in biological isolated neurons, might be rather explained by the natural biological variability and noise [36] that act by mixing different regimes of oscillation.

If the best CPGs are those made of model neurons tuned at the transition from periodic bursting to tonic spiking, where a rich variety of dynamical behaviors are found, variability and noise are able to mix several windows of periodic behavior, spike-adding transitions, and chaotic windows, providing an irregular time-series that is comparable to the ones from biological isolated neurons.

The main question, however, might be the biological meaning underlying these findings, which suggest that neurons are in a very particular dynamical state, i.e., in a narrow range in the transition between two distinct modes of operation (bursting and tonic). Hence, such delicate balance must be sustained by a sort of homeostatic process that keeps these neurons in such state. In one hand, there are experimental evidences that general dynamical properties can be maintained even with disparate individual features [22]. On the other hand, evidences suggest that from different individuals neurons can present very different parameters and yet, keep the same dynamical properties [33, 61]. These evidences, along with our results, suggest that there might be a “target dynamics” instead of a target parameter, in which a particular mode of operation is maintained. The way the phisiological mechanisms act to maintain such balance is still to be uncovered.
